# A Model for Out-of-Hospital Multispecialty Emergency Medicine: Accomplishments and Challenges

**DOI:** 10.1177/1178632918805996

**Published:** 2018-10-23

**Authors:** George Theocharis, Konstantinos S Kechagias, Michael Oikonomou, Stamatia Chorepsima, Dionisis Rodis, Ioannis Salpigktis, Matthew E Falagas

**Affiliations:** 1 SOS Doctors, Athens, Greece; 2 Department of Medicine, Euroclinic General Clinic, Athens, Greece; 3 Alfa Institute of Biomedical Sciences (AIBS), Athens, Greece; 4 Department of Gastroenterology, Euroclinic General Clinic, Athens, Greece; 5 Department of Medicine, Henry Dunant Hospital Center, Athens, Greece; 6 Department of Medicine, Tufts University School of Medicine, Boston, MA, USA

**Keywords:** Out-of-hospital services, medical needs, Greece, home visit

## Abstract

Home care has been traditionally considered as an important type of medical service. “SOS Doctors” is a Greek organization providing out-of-hospital multispecialty emergency medicine services the past 25 years. Its services mainly meet the demands of the elderly and the nonambulatory patients. The decreased number of hospitalizations, hospital-related infections, and need for patient transportation are the main advantages of a model for out-of-hospital multispecialty emergency medicine. However, the time consumed by the doctor related to transportation is a drawback of medical house calls. Despite the challenges, medical house calls are a useful part of health services in the modern health care system.

In the past, medical care was inextricably linked with medical house calls. In emergency cases, the doctor was present at the patient’s bedside to offer his medical service.^1^ Over the years, progress in the medical field, lifestyle changes for both patients and clinicians, and urbanization have led to a massive decrease in the number of medical house calls. This decrease led to the consolidation of hospital-based medical care systems.^2^

Moreover, the working habits of doctors have also changed in recent years. The doctors’ rhythm of work has been intensified, with most of their hours being spent in the hospital and the medical office. To meet a target income or generate a target profit for a firm, the time allowed for providing care by physicians for each patient has shrunk dramatically. Medical house call, as a process, is also time-consuming and costly for the doctors because of the transportation and as a result physicians’ productivity regarding the number of patients able to visit is reduced. In addition, economic crisis and specifically increased taxation incommode the function of private medical house call organizations. These facts constitute major discouraging factors to offer this kind of medical service.^3^ Thus, the number of doctors who perform medical house calls has been gradually reduced.^4^

The progress of medical science has contributed to a significant increase in the average life span and the expected survival of patients with chronic and incurable illnesses. Thus, more patients nowadays such as elderly, people with chronic illnesses, or other health-related problems and people with limited mobility or high risk of falls could benefit from medical care provided at their own home.^5^ Therefore, an integrated medical health system should also include medical house call services.

Also, the increased specialization in the medical field led to the fragmentation of the basic medical specialties. As a result, the traditional “one patient-one doctor” relationship has been turned into a “one patient-many doctors” relationship. The elderly have different health problems and each doctor deals with the ones that are relative to his or her specialty and do not adopt a holistic patient care approach. In this perception of medicine, the model of “SOS Doctors” has been created to be a potential means for personalized medical care by specialized physicians.

“SOS Doctors” is a private organization founded in February 1993 in Athens based on the model of medical health service originated in Paris, in 1966 with the name “SOS Médecins”, which today offers its services to approximately one-third of the population in 32 cities in France. The Greek “SOS Doctors” uses a model with expanded services that include specialized physicians and equipment (ultrasound, Xrays, etc.).

The model of “SOS Doctors” fits especially well with the needs of elderly and nonambulatory patients, for whom the hospitalization may be more harmful than beneficial due to multiple reasons, including the in-hospital infections as well as the abolition of their self-image and self-estimation.^6^ The diagnostic evaluation of these patients in a hospital basis is often impossible due to difficulties regarding their transportation.^5^ House calls allow these people to remain in their home or other residential setting and avoid the risks discussed above.

Home hospitalization and palliative care constitute a significant part of current medicine due to the aging population. Elderly and nonambulatory patients need a constant access to a health service 7 days a week and 24 hours a day.^7^ The priority of a health system providing medical house call services is to have a continuous supervision of the patient’s health condition by knowing his or her medical records and also guide the patients into the health systems which are becoming more and more complex nowadays.

Moreover, “SOS Doctors” or similar organizations may provide substantial support to primary care. Data from a retrospective analysis,^8^ conducted between January 2005 and December 2015, regarding the demand of physician house calls in Greece, showed that despite the ongoing economic crisis, the request of house calls was not decreased. Specifically, during an 11-year period, “SOS Doctors” performed 335 123 house calls. The peak of these medical visits was observed in 2009, with a gradual fall during 2010-2013 and stabilization the next 2 years. The integration of radiology in the provided services of “SOS Doctors” led to an increasing demand, starting at 352 calls and reaching 2230 calls during a 6-year period (2009-2016).^8^

Regarding the financial aspects, a model like “SOS Doctors” can be adopted by countries where patients pay their doctors and can be easily combined with collaboration with private clinics to achieve continued care. It can also be used by insurance companies to reduce costs by reducing unnecessary hospitalizations.^9^ Indeed, according to the data from the meta-analysis discussed above, although most of the house calls were performed for acute medical conditions, only 9.2% of those patients were advised for admission to the hospital. The most common medical condition was lower and upper respiratory tract infection (19.4%), followed by cardiovascular diseases (10.3%), musculoskeletal (9.1%), gastrointestinal (6.3%), and neurological disorders (3.7%).^6,8^ According to the current data, the elderly and female patients requested more house calls than male and younger patients and most of the phone calls were performed between 4 and 12 pm.^6,8^

The aging population needs services that cannot be provided exclusively from the current structures of the national health care system. The integration of services of organizations such as “SOS Doctors” in the medical care of elderly and nonambulatory patients may result in a better quality of life for this subpopulation.

In Greece, “SOS Doctors” offer their services mainly in Athens and Thessaloniki. The efforts for expanding the organization to the rural regions should be intensified because the satisfaction rates of the patients who have used the provided services are high (97.5%). The higher satisfaction rate (98.1%) was noticed in patients 76 to 90 years old and the lower (97.3%) in patients 30 to 45 years old. The most common reason for lack of satisfaction was the doctor’s behavior, regarding the time spent to examine and discuss the medical problem with the patient.^8^ These data are consistent with the general perception that the patients need the attention and the continuous care of specialized doctors. The problems, discussed above, regarding the technical difficulties of diagnostic and therapeutic evaluation of fragile patients, are even more obvious in smaller cities and villages.

Models for out-of-hospital medical services offered by specialized doctors, including the one used by the organization of “SOS Doctors” may tender valuable help in patients. The current economic crisis in Greece has led to additional challenges in the function of “SOS Doctors”, but the persistent efforts of the organization offer the chance of a meliorated medical health care to patients.
